# Magnetic resonance imaging

**DOI:** 10.1186/1546-0096-12-S1-I23

**Published:** 2014-09-17

**Authors:** Clara Malattia

**Affiliations:** 1 Pediatrics, University of Genova, Italy; 2 Peditria II Reumatologia, Istituto Giannina Gaslini, Genova, Italy

## 

MR imaging is encountering an expanding application in the assessment of patients with paediatric chronic rheumatic diseases. By providing multiplanar tomographic imaging with unparallel soft tissue contrast, MRI allows the simultaneous evaluation of all joint structures involved in inflammatory arthritis and it is regarded as one of the most attractive imaging modalities for the investigation of juvenile idiopathic arthritis (JIA). MRI provides additional and more sensitive information over clinical examination and other imaging modalities and holds great promise in supporting diagnosis of JIA, assessing its severity and prognosis, monitoring disease course and treatment efficacy.

The use of MRI in the assessment of the musculoskeletal system in children has important differences from its adult counterpart. Growing joints change anatomically over time making imaging in JIA a real challenge without the availability of normative data. A sound knowledge of growth-related changes, in fact, is of foremost value to establish whether joint surface changes reflect a real damage or are actually part of normal development.

Main indications for musculoskeletal MRI in paediatric rheumatology, technical issues and diagnostic accuracy, pitfalls in image analysis and newer MRI and scanner techniques (i.e whole-body MRI) will be discussed thus providing an overview of the recent advances and challenging in imaging children with rheumatic disorders.

## Disclosure of interest

None declared.

